# EXPLORING RACIAL DIFFERENCES IN WILLINGNESS FOR COGNITIVE TESTING IN OLDER ADULTS

**DOI:** 10.1093/geroni/igae098.3271

**Published:** 2024-12-31

**Authors:** Amy Costa, Whitney Kasenetz, Alex Crisci, Ashley Curtis, Jennifer O’Brien

**Affiliations:** University of South Florida, Tampa, Florida, United States; University of South Florida, Tampa, Florida, United States; University of South Florida, Tampa, Florida, United States; University of South Florida, Tampa, Florida, United States; University of South Florida, Tampa, Florida, United States

## Abstract

Knowledge and attitudes about dementia contribute to diagnosis and treatment outcomes. People of color (POC) are less likely to receive dementia screening and an official dementia diagnosis compared to white adults, leaving them vulnerable to missed diagnosis and early intervention. This study tested whether racial group (0=white, N=255; 1=POC, N=56) moderates associations between knowledge about Alzheimer’s disease (AD) and willingness to undergo cognitive testing. Older adults (N=364, Mage=69.5±6.3 years, 171 women) completed the Perceptions Regarding Investigational Screening for Memory (PRISM; acceptance of AD screening sub-score [PRISM-accept]) and the Alzheimer’s Disease Knowledge Scale (ADKS). After completing the questionnaires, participants were invited to the lab to complete the Montreal Cognitive Assessment (MoCA; 0=not completed, 1=completed). A linear regression determined whether race moderated associations between ADKS and PRISM-accept sub-score, controlling for education and gender. A logistic regression determined whether race moderated associations between ADKS and MoCA completion. Race did not moderate associations between ADKS and PRISM-accept (p>.05). Race moderated associations between ADKS and whether they completed the MoCA (χ2=12.2, p<.001). Specifically, higher knowledge about AD had higher odds of MoCA completion for white older adults (B=.10, p<.001), but lower odds of MoCA completion for POC (B=-.21, p=.03). Results reveal increased knowledge about AD may increase willingness to be screened for white older adults but decrease willingness for POC. Results may be due to factors influencing screening acceptance in POC (e.g., lack of trust). The present study highlights the need to tailor recommendations to increase willingness to be screened for dementia based on race.

